# Investigation on Reliability and Scalability of an FBG-Based Hierarchical AOFSN

**DOI:** 10.3390/s100402901

**Published:** 2010-03-29

**Authors:** Li-Mei Peng, Xin-Wan Li, Won-Hyuk Yang, Young-Chon Kim

**Affiliations:** 1 School of Information Technology, Chonbuk National University, Jeonju 561-756, Korea; E-Mails: aurora_plm@hotmail.com (L.-M.P.); k0kok0@hotmail.com (W.-H.Y.); 2 GRID Middleware Research Center of ICC, Korea Advanced Institute of Science and Technology, Taejon, 305, Korea; 3 The State Key Laboratory of Advanced Optical Communication Systems and Networks, Department of Electronic Engineering, Shanghai Jiao Tong University, Shanghai 200240, China; E-Mail: lixinwan@sjtu.edu.cn

**Keywords:** AOFSN, FBG, hierarchical, reliability scalability

## Abstract

The reliability and scalability of large-scale based optical fiber sensor networks (AOFSN) are considered in this paper. The AOFSN network consists of three-level hierarchical sensor network architectures. The first two levels consist of active interrogation and remote nodes (RNs) and the third level, called the sensor subnet (SSN), consists of passive Fiber Bragg Gratings (FBGs) and a few switches. The switch architectures in the RN and various SSNs to improve the reliability and scalability of AOFSN are studied. Two SSNs with a regular topology are proposed to support simple routing and scalability in AOFSN: square-based sensor cells (SSC) and pentagon-based sensor cells (PSC). The reliability and scalability are evaluated in terms of the available sensing coverage in the case of one or multiple link failures.

## 1. Introduction

Optic fiber sensor networks, which are multiplexed with arrays of optical fiber sensors, have received increasing attention due to their attractive advantages. They are immune to electromagnetic interference, harsh or hostile environments, and therefore can be deployed in areas where electrical-based sensors would fail or require expensive protection. A number of similar or different sensors can be attached along a single optical fiber, and remote data over kilometers can be processed without corruption. Among the optical fiber sensors, Fiber Bragg Grating (FBG) sensor has been the most attractive type due to its smart architecture, large-scale multiplexing capability, immunity to electromagnetic interference, and because it is power-free. A popular technique for multiplexing FBG sensors is wavelength division multiplexing (WDM). The number of accommodated FBG sensors is determined by the usable spectral bandwidth of the system and the wavelength-shift of each FBG sensor. FBGs have been studied extensively in terms of strain and temperature measurements. Because of the wavelength-encoded nature of FBGs, they can be wavelength-division multiplexed to form an array or network for multi-point or quasi-distributed measurements. A popular scheme to realize a WDM FBG sensor network involves using a tunable optical filter (TOF) and detecting the peak wavelengths when the TOF is scanned through the FBG spectrums in [1–4].

The applications of FBG based AOFSN are diverse, such as in environment monitoring, home caring, *etc*. Another application, called structural monitoring, is becoming very important as ever more high buildings, large mansions and huge bridges are built. Structural monitoring for such as tunneling, building and bridge health are very important, because their damage or collapse will cause serious accidents. In order to monitor their security status, large-scale sensor networks are necessary and the FBG based AOFSN becomes an appropriate candidate. Regular arrays of FBG sensors multiplexed into fibers are distributed inside them to construct large-scale AOFSN and monitor their construct security. Such kind of large-scale AOFSNs require high reliability to guarantee the monitoring accuracy.

Many researches that consider the reliability or construction of sensor networks based on FBG have been studied. A novel fiber-laser-based sensor network with a self-healing function is proposed in [1]. It is based on adding switches to self-healing ring architectures. Some novel designs of wavelength multiplexed fiber sensor networks that are tolerant to one or more cable failures are proposed in [2]. They used protection switching to recover service. In [5], the authors introduce a low cost array which has a low susceptibility to failure when damage is induced for smart structures using FBGs as sensing elements. In [3], a star-bus-ring architecture for FBG sensors was proposed. The FBG survivability and capacity of a multipoint sensor system are enhanced by adding remote nodes and 2 × 2 optical switches. Research [4] proposed a new sensor-network model that considers the survivability and expansibility. However, complex switches were used to ensure reliability, which increases the cost and SNR.

Based on the above achievements, we provide a further detailed insight into the construction of a large-scale AOFSN network using FBGs. The proposed AOFSN is composed of three levels and can be classified into two active levels consisting of the first interrogation level and the second RN level, and a passive level consisting of FBGs. The reliability of the AOFSN network is improved by achieving reliability in the second and third levels. As the second level consisting of RNs is active, reliability is realized by proposing switching architectures for RN nodes. This enables the optical signals to be switched through different routing paths in order to recover FBGs distributed besides failed links. The FBG based AOFSN can construct power-efficient sensor network, as the passive FBG requires little power to control, but requires complex and expensive interrogator systems to collect and analyze the sensing signals. Therefore, simple but efficient routing schemes are necessary to reduce the use of interrogations and to alleviate the burden of the limited and expensive interrogations. As the proposed AOFSN network is mainly constructed by wired and passive FBG sensors, the routing scheme aims at constructing unicursal and regular virtual topologies. The unicursal concept enables us to scan all or near to all FBG sensors by emitting light once. The regular concept enables the interrogations to control and process the sensing signals easily. The polygon cell based AFOSN is considered to be an appropriate candidate, as they remain unicursal and regular after being extended. More specifically, the square and pentagon based AOFSN will be discussed in our research as examples of polygon-based topologies for AOFSN.

In this paper, we first review the proposed different kinds of hierarchical AOFSN networks in our previous research in [6]. The two regular types of sensor cells (SC) called the square-based SC (SSC) and the pentagon-based SC (PSC), and how to improve the reliability and scalability through the third passive SSN level are all reviewed firstly. Then, the reliability and scalability are numerically considered in this paper. The reliability is evaluated in terms of how many link failures the proposed AOFSN can tolerate. The scalability is evaluated in terms of whether a scaled sensor network can maintain a self-similar virtual structure with its lower level structure, so as to maintain a similar sensor routing scheme. More exactly in this paper, the reliability is calculated and evaluated by considering the coverage sensing area when one or multiple link failures happen, and the scalability is calculated and evaluated by considering the number of required FBGs, the cost of the required number of 2 × 2 switches and the corresponding coverage.

### 2. Hierarchical AOFSN Architectures

As mentioned above, the proposed AOFSN comprises three levels: the first is the interrogation/sever (InS). It manages the second and third levels to check for link failure between RNs and InS or sensor cells, by sending and collecting scanning signals from RNs; the second level is the interface/RNs (Remote Nodes). Several RNs comprise the self-healing ring architecture. Each RN manages its own sensor subnet to collect scanning signals from the third level and then they provide feedback to the first InS level. The third level is the sensor subnet. It consists of some passive FBG sensors, executing the scanning request and providing feedback to their upper RN level.

**Figure 1.**
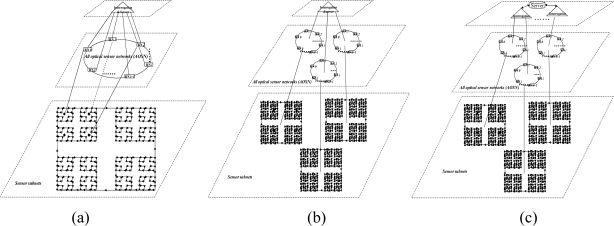
**(a)** No grouping in the first two levels; **(b)** Grouping in the second level; **(c)** Grouping in the first and second levels.

The cost of hardware in AOFSN is mainly due to the interrogation and switches and the interrogation is the most expensive device. In order to reduce the cost of the most expensive interrogation device and utilize it efficiently and sensibly, three different types of hierarchical AOFSNs are proposed. They mainly differ in terms of whether the second communication network level and the first interrogation level are grouped. By grouping them, the cost due to interrogation and the burden of interrogation for demodulating large numbers of signals can be reduced in order to utilize resources efficiently. The architectures are discussed in detail and shown in Figure 1.

The first type is shown in Figure 1. The first level is composed of one interrogation/server for signal recognition. The second level is the interface level between the interrogation/server and sensor subnets. It is composed of a self-healing unidirectional ring consisting of *N* RNs. Each RN manages its own sensor subnet. All RNs are centrally controlled by the only interrogation and are responsible for collecting data and providing feedback to it. Thus, the RNs can simultaneously receive the scanning requests from a common interrogation, and they execute the scanning process to provide feedback of the scanning results to the interrogation. Finally, the interrogation selects the signals useful for recognition and decides the sensing results from all the feedback signals by the below RNs. The second type is shown in Figure 1(b). Of all the sensing signals collected by the RNs in the second level, the useful ones can be quite rare. Many researches on wireless networks apply a three-point location mechanism. Only three sensor nodes are activated for sensing and they provide useful sensing signals each time. In the AOFSN, we apply a similar candidate mechanism in the second level in order to reduce the burden of the only interrogation. That is, the RNs in the second level are grouped according to their geographical positions instead of providing all the raw sensing signals to the only interrogations directly. Each time the interrogation sends the scanning request to its below RNs, it estimates the approximate geographical scanning region from the results of the last scanning process. Then it first selects one or several specific groups to do the scanning process and collects the feedback signals from these specific groups. Thus, the burden of the interrogation is further reduced and the scanning speed is improved. This screen-out mechanism can be realized by selecting nearby regions with higher strength signals according to the last scanning results. The third type is shown in Figure 1(c). It is an alternative from the second type. The interrogation level is further grouped according to the interrogating techniques. This architecture is applicable for heterogeneous sensor networks, as each interrogation can interrogate different types of feedback scanning signals. This type has high flexibility compared to the first two types but there is a higher cost, because more interrogations are used. The three types of hierarchical architecture were proposed based on the cost or burden of the first two levels. The construction of the SSN will be discussed in the next section.

## 3. Sensor Subnet Construction

FBG has been one of the most promising sensor technologies and has been dynamically developed during past decades. FBG is known for its passive characteristic, *i.e.*, FBG sensors are passive devices and the transmit carrier involves passive sensing signals. The signals are sent and reflected passively without any active actions of their own. The sensing action is processed via InS (or RNs) in the higher levels, by comparing their reflected wave or other parameters with their original ones. Therefore, for such a wired and passive sensing system, consideration of a unicursal- and regular- connected network topology is necessary. The unicursal concept is suggested in order to guarantee that all the FBG sensors can be scanned at once. This enables us to multiplex as many FBGs as possible in one fiber in order to reduce the cost. However, to increase the reliability of the AOFSN, several 2 × 2 switches are needed to do the recovery process when link failure happens. The regular concept is suggested in order to provide a simple routing scheme and improve the scalability. A regular topology is always easy to route and can be scaled to larger ones while maintaining a self-similar virtual regular topology and a self-similar routing scheme. Based on the above considerations, this section proposes two main basic virtual topologies based on the square-based SC (SSC) and pentagon-based SC (PSC), respectively. As we previously mentioned, the third level is further divided into three levels, denoted by the Sensor Cell (SC), Sensor Subnet Group (SSG) and Sensor Sub Network (SSN).

### 3.1. Two Unicursal and Regular SC-Based AFOSN

#### 3.1.1. Square SC (SSC)-Based AFOSN

**Figure 2.**
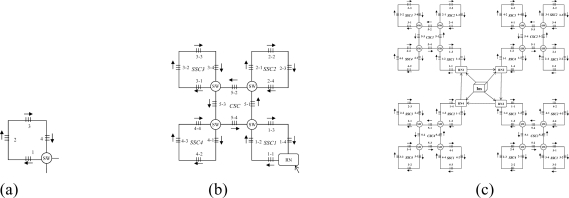
**(a)** SSC; **(b)** SSC group; **(c)** SSN.

Firstly, we apply a square model as a sensor cell to construct the sensor subnets. As shown in Figure 2(a), four FBGs (denoted 1–4) are distributed on each side of the square and one 2 × 2 switch is set at the joint point of FBG 1 and FBG 4 in order to guarantee the reliability of its SSC. It should be mentioned that more FBGs can be set on each side of an SSC, which can also be seen as a virtual ring topology. For simplicity, we discuss the SSC topology which has only one FBG set on each side as shown in Figure 2(a).

The 2 × 2 switch is very important for the SSC in view of survivability if any link failures happen, as described in [3,6]. Light can be sent in and out of any of the four ports. The scanning direction is clockwise in the normal case and counterclockwise in case of link failure. The SSC can be easily scaled to a larger SSC group (SSCG) by connecting four SSCs and the SSCG will still maintain a square-like virtual topology, as shown in Figure 2(b). One more FBG is set on the link that connects two SSCs. Therefore, 20 FBGs are embedded after the first scaling. In other words, each SSCG consists of four SSCs and one CSC (central SC). The SSCG can be further scaled to a larger group, which consists of four SSCGs and one CSC. The detail is discussed in the following section. A sensor subnet is composed of one or several SSCGs, and we give an example of an AOFSN with an SSN consisting of four SSCGs, as shown in Figure 2(c).

As mentioned in Section 2, there is one interrogation and four RNs, and the interrogation is connected to all four RNS in order to enable light to be sent to them for scanning. The four RNs are constructed in a ring manner, thus each is also responsible for recovery when failure happens between their upstream RN and the interrogation. In the normal case, the optical signals are sent from the interrogation to the four RNs; and then, each RN sends the signal to its responsible SSCG in the clockwise direction, in order to collect the feedback signals from all the FBGs. After collecting the feedback signals, the RNs analyze if there is any change of the sensing signals. If so, the RN nodes provide the feedback of this change to the interrogation; otherwise, they don’t provide any feedback signal in order to save the energy of the interrogation.

#### 3.1.2. Pentagon *SC (PSC)*-Based AFOSN

**Figure 3.**
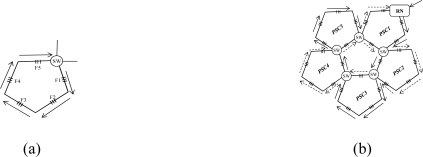
**(a)** PSC; **(b)** PSC group (PSCG).

This section considers a pentagon-based sensor cell to construct each sensor subnet. Similar to a SSC, five FBGs (denoted 1–5), are distributed on each side of the PSC as shown in Figure 3(a). Similarly, one 2 times; 2 switch is set at the joint point of FBG 1 and FBG 5 in order to guarantee the reliability of each PSC. Light can be sent in and out of any of these four ports. In the normal case, the scanning direction is clockwise, and vice versa. PSCs can also be easily scaled to a larger PSC group (PSCG) by connecting five PSCs, as shown in Figure 3(b). Two PSCs share a switch and the PSCG also maintains a pentagon-like virtual topology for further scaling. A sensor subnet is composed of one or several PSCGs, which is similar to that of the SSCG. The difference with the SSC is that no more connected links are needed when PSCs are scaled to larger PSCGs. The reliability of the higher two levels is discussed and realized mainly by resetting the switch in the RN. Thus, the switch architectures are designed first and then the reliability of the SSN level is discussed.

## 4. Consideration of Reliability

### 4.1. Switch Architecture for Higher-Level Reliability

**Figure 4.**
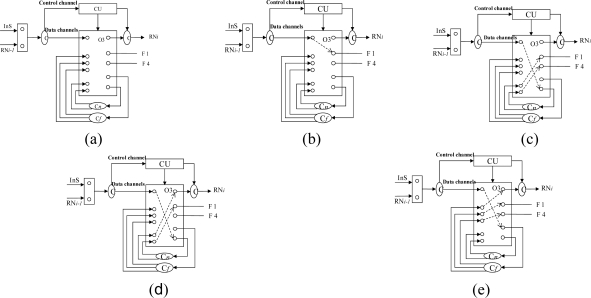
**(a)** Switch architecture of RN; **(b)** Normal case; **(c)** Failure in level 3; **(d)** Failure between level 1and 2; **(e)** Failure between level 1 and 2, within level 3.

There are two kinds of link failures of the first two levels: the failure between the interrogation and RN, and the failure between RNs. Considering Figure 2(c) for example, only one link failure between two RNs does not affect the scanning process for each SSN. However, if a link failure occurs between the interrogation/server (InS) and RN, the RN cannot receive the emitted scanning light from the InS and the whole SSN under the charge of it cannot be scanned and detected. To solve this problem, RNs are connected in a unidirectional ring manner in order to perform self-healing in case of a link failure between itself and InS, as shown in Figure 2(c). In that case, the RN receives scanning signals from its upstream RN nodes instead, so as to guarantee good reliability. Therefore, the switch architecture within the RN nodes is crucial for the recovery process and is shown in Figure 4(a).

**Figure 5.**
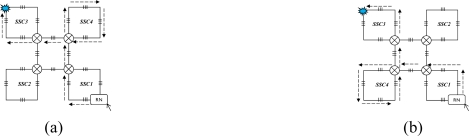
**(a)** Single-link failure; **(b)** Self-healing for one link failure.

Four couplers are needed, two for discriminating the control signal from the scanning signals (SSs); the other two couplers, named C*n* (1 × 2) and C*f* (1 × 3), are used for recovery when a link failure happens. In the normal case, considering Figure 5 for example, the RN receives light from the interrogation and then emits light from FBG 1-1 to the other FBGS in a clockwise direction in order to collect SSs, as shown in Figure 4(b); if a link failure happens in the third level, signals are emitted from InS and split into two by switching to C*n* (1 × 2) in order to scan from F1 and F4 simultaneously, as shown in Figure 4(c). While in case of a link failure between RN*_i_* and InS, the switch in node RN*i*−*1* will be reset as follows: scanning signals emitted from the InS port are switched to C*n* (1 × 2) and split into two, one for its own SSN from F1 and one for the input of RN*i*, as shown in Figure 4(d). If there is a link failure between RN*_i−1_*’s downstream node and InS, and a simultaneous link failure within its own managed SSN, the switch is setup as shown in Figure 4(e) for node RN*_i−1_*, and scanning signals received from InS are switched to C*f* (1 × 3) and split into three, two for its own SSN’s scanning from both sides and one for the input of RN*_i_*. In view of node RN*_i_*, it receives the scanning signal from the input port RN*_i−1_* instead of InS.

### 4.2. Reliability in SSN Level

This section discusses the reliability of the SSN level for both SSC- and PSC-based AOFSN in terms of their self-healing abilities. Considering the SSNs shown in Figure 2(b) and Figure 3(b), both are tolerant to at least one link failure. If more than one link failures happen, some of the 2 × 2 switches should be selected for resetting according to the failure point. Figures 5(a) and (b) show the case of one link failure in region SSC3. In the normal case, light is emitted from the RN which is connected to SSC1 and then the RN sends scanning signals in a clockwise direction to the remaining sensors. When one link failure happens, as shown in Figure 5(b), the switch in the RN is set to that shown in Figure 4(b), and the RN emits light simultaneously in both the clockwise and counterclockwise direction for recovery.

**Figure 6.**
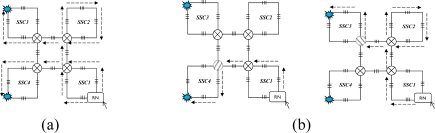
**(a)** Dual-link failures; **(b)** Self-healing for dual-link failures.

**Figure 7.**
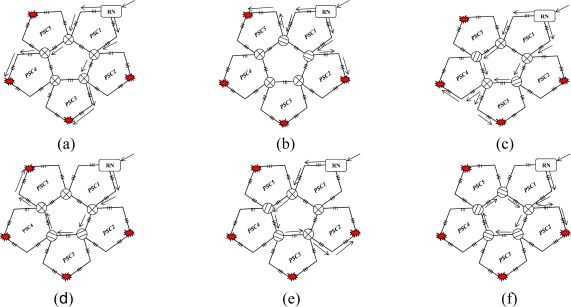
**(a)** Four link failures; **(b)** Self-healing 1; **(c)** Self-healing 2; **(d)** Self-healing 3; **(e)** Self-healing 4; **(f)** Self-healing 5.

In case of two link failures as shown in Figure 6(a), two sensors in SSC3 and SSC4 cannot be scanned in the normal case. In order to rescan the remaining separated sensors, the 2 × 2 switches in the two regions SSC3 and SSC4 are reset as shown in Figure 6(b). The switch in the RN is also reset in Figure 4(b). The reset signal was previously transmitted through the control channel. The recovery process for the SSN of PSC-based AOFSN is shown in Figures 7(a)–(f). The PSC-based SSN in Figure 3(b) can be made tolerant to up to four simultaneous link failures by resetting the 2 × 2 switches within each PSC. There is a separate control channel similar to that of the switch architecture of the RN. The control signals for switching settings were sent previously through the control channel before sending the scanning signals, so as to set up the switches, then the scanning signals are resent for self-recovery, as shown in Figure 7. Figure 7 shows the case of four link failures, which is the extreme case, *i.e.*, even though the case of simultaneous four failures will rarely happen, we provide the example to describe the self-healing process showing the reliability of the PSC-based SSN. This kind of PSC-based SSN can achieve high reliability with strong self-healing capability, by using and resetting several 2 × 2 switches. The case of two simultaneous link failures in the same region can also be tolerated for the PSC-based SSN. In order to recover from the link failure in time, the InS emits light to detects the link failure periodically. Each time when InS emits ligth to detect the link failure, it considers there is no link failure and sends signals to set all the switches as shown in Figure 4(b). The switching time of each switch is supposed to be δ and the total(longest) fiber length is set to be *l*. Therefore, the timeout time for the InS to wait for the feedback signals is $T_{\mathrm{timeout}}=l/v+\sum_{j=0}^{s}\delta_{j}+\alpha$, where *v* is the velocity of light speed in fiber, *s* is the total number of switches, and *δ_j_* is the switching time of the j-th switch, α is the extra time to guaratee that InS can receive the feedback signal. If InS receives no feedback signal after T*_timeout_*, InS will judge that all the links between InS and RNs are broken. On the other side, if all the signals are feedbacked after T*_timeout_*, InS decides there is no link failures anywhere. Otherwise, if any emitted signals are not feedbacked, InS can decide the positions of failure links and their responsible switch, and then start to recover. The recovery signals are decided by the InS, distributed to RNs, and executed by switches as introduced in Chapter 4.

## 5. Consideration of Scalability

**Figure 8.**
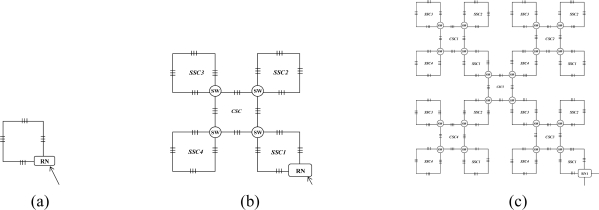
**(a)** SSC; **(b)** first extension; **(c)** second extension.

**Figure 9.**
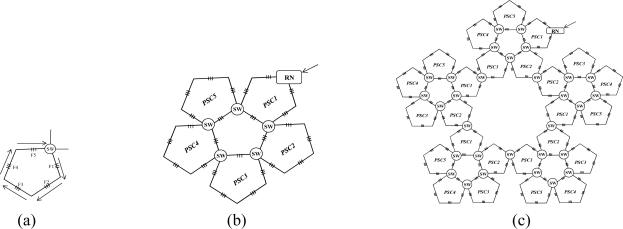
**(a)** PSC; **(b)** first extension; **(c)** second extension.

This section discusses the scalability of the proposed AOFSN. Large scale sensor networks consisting of hundreds of thousands of sensor nodes can link the physical world to global communication networks for a broad set of applications. The current technology for embedding FBG sensors can multiplex as many as 1000 FBGs into one fiber in order to construct a large sensor array. Considering this advantage and the current requirement for large-scale sensor systems, it’s necessary to discuss the performance of our proposed SSN after scaling to a larger scale. As we have discussed, for the small-scale SSNs, which consist of the 20-FBG shown in Figure 5 for SSC, and the 25 FBGs shown in Figure 8 for PSC, we tried to scale them in order to multiplex as many as possible. After scaling the small-scale SSNs to a large-scale AOFSN system, the maintenance of the routing mechanisms for scanning sensors, in both the normal case and the case of link failure happening, is important for maintaining low cost and simplicity. The SSC- or PSC-based SSNs were proposed to realize scaling, as previously mentioned. As their topologies are regular and unicursal, they have good scalability in view of maintaining a self-similar virtual topology and the same recovery routing algorithms for the much larger scales shown in Figures 8 and 9. As we can see in Figure 8, Figure 8(a) shows the basic square-based sensor cell (SSC) consisting of four FBGs and an RN node. Figure 8(b) is scaled from the SSC shown in Figure 8(a) and it is consisted of four SSCs and an additional central sensor cell (CSC). The CSC in the center of the four SSCs is added to connect the four SSCs, and its architecture is the same as the other SSCs. Figure 8(c) is scaled from the SSC group (SSCG) shown in Figure 8(b) and is consisted of four SSCGs and an additional CSC5 in the center to connect the four SSCGs. By scaling in the manner shown in Figures 8(b) and (c), the routing mechanism in the normal case and in case of link failures can be maintained, as the architectures in Figures 8(a), (b) and (c) remain self-similar. The differences are that more switches should be set for the high-scale architectures, to switch the control signals in case of link failures.

In view of the PSC-based architectures shown in Figure 9, the process is very similar to that shown in Figure 8. The architecture in Figure 9(b) is scaled from that shown in Figure 9(a) and consists of five PSCs. Similarly, the architecture shown in Figure 9(c) is scaled from that shown in Figure 9(b) and consists of the five PSCGs shown in Figure 9(b). Two adjacent PSCGs are connected by one switch, thus improving the reliability. So, we propose the concept of scaling degree. The scaling degree is one based on Figures 8(a) and (b), and it is two based on Figures 8(b) and (c).

## 6. Numerical Results

### 6.1. Reliability Analysis

**Figure 10.**
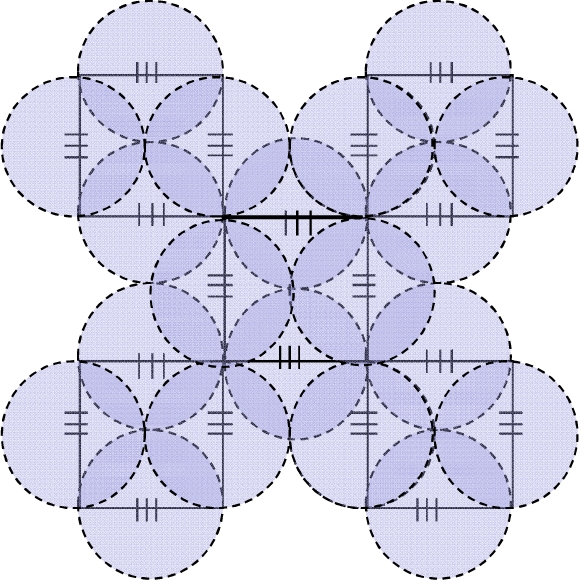
Coverage of SSC group (SSCG).

The reliability of a sensor network in case of node or link failures is crucial, as it decides the sensing accuracy of an objective in case of failures; node or link failures do happen as time advances. The sensing accuracy of a sensor network is further decided by the coverage of the sensors. In other words, the sensing coverage characterizes the monitoring quality provided by a sensor network in a designated region. Distributed detection requires that every location be monitored by one or multiple nodes, and distributed tracking and classification requires even higher degrees of coverage. A network with a higher degree of coverage can maintain acceptable coverage in the face of higher rates of node or link failures. So the coverage of sensors in case of failures also decides the reliability of the sensor network. In this research, the sensing range of all the FBG sensors is assumed to be *r* and the length of the square side is supposed to be 2*r*. Moreover, the sensing range of each FBG sensor is supposed to be represented by a circle, considering the FBG as the central angle and *r* as the radius, as shown in Figure 10. 24 FBGs are used in the SSC group (SSCG) and the total coverage of the 24 sensors is shown by the circles in shadow. The coverage area of the 24 FBGs is calculated by formula (1).

(1) $$S_{\mathrm{Covered}}=5\times {\left(2r\right)}^{2}+8\pi r^{2}+4\times \left(\frac{4r^{2}-\pi r^{2}}{2}\right)$$

In order to calculate the reliability of the proposed AOFSN architecture, the coverage area when a link failure happens needs to be considered. The probability of a fiber link failure is supposed to be *p*. Thus, the probability of one link failure within one SSC in Figure 8(a) is calculated by formula (2), where *n* is the number of FBGs within a SSC (four in this case). It is assumed that more than one link failure within one SSC cannot happen, but multiple link failures within different SSCs can happen. Consider Figure 8(b) for example; it consists of five SSCs, so the probability of *i* simultaneous link failures, *P_i_*, is calculated by formula (3), where *N* is the number of SSCs (five in this case).

(2) PSSC=Cn1p1(1−p)n−1

(3) Pi=CNiPi(1−PSSC)N−i

Then, the lost coverage after *i* link failures has happened is calculated for the SSC-based network. In section 4.1 and 4.2, reliability has been achieved by proposing the recoverable switching RN node. The recovery paths according to the proposed switching nodes have also been shown. However, there are still some FBGs that cannot be recovered if more than one link failure happens. If an FBG is not recovered, it loses its sensing coverage. Consequently, the reliability of the SSC-based network is affected. It is calculated by formula (4), where *A_i_* is the sensor network availability when link failure happens and *R_Lost_(i)* is the loss rate of sensing coverage when *i* link failures have happened.

(4) $$A_{i}=1-P_{i}R_{\mathrm{Lost}}(i)$$

#### (1). One link failure

When one link failure happens, the FBGs separated by the failed link can be recovered by resetting the switches appropriately in the RNs, as shown in Figures 5(a) and (b). Thus, there will be no loss in the sensing coverage area and the AOFSN network can still operate as normal, as there is no link failure. Moreover, wherever the one link failure happens, *i.e.,* regardless of whether it is in SSC1, SSC2, SSC3, SSC4 or the central sensor cell, the sensor network can recover the link failure by routing to the lost FBGs through different paths. In conclusion, the coverage loss ratio is zero when only one link failure happens, *i.e.*, *R_Lost_(1)* = 0. The availability when one link failure happens is calculated by formula (5) according to (4) and is 1 or 100%.

(5) $$A_{1}=1-P_{1}\times R_{\mathrm{Lost}}(1)=1$$

#### (2). Two link failures

**Figure 11.**
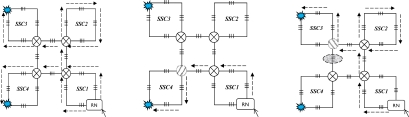
Unrecoverable FBGs in case 1.

**Figure 12.**
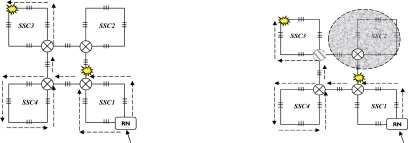
Unrecoverable FBGs in case 2.

When two link failures happen, most of the FBGs separated by the failed points can be recovered by resetting the switches in the RNs and the 2 × 2 switches that connect two sensor cells. However, not all FBGs can be recovered, *i.e.,* some sensing coverage will be lost. Moreover, the lost coverage depends on the positions of the failed links. There are various combinations of positions of the two link failures, *i.e.*, they may lie in SSC1 and SSC2, or SSC2 and SSC4, or between the central SSC5 and any other SSCs. Different combinations lead to different lost coverage. Thus, the reliability of two link failures cannot be calculated directly according to formula (4). After analyzing the lost coverage of all combinations, they can be divided into the two cases shown in Figures 11 and 12.

***Case 1:***

***Case 2:***

**Figure 13.**
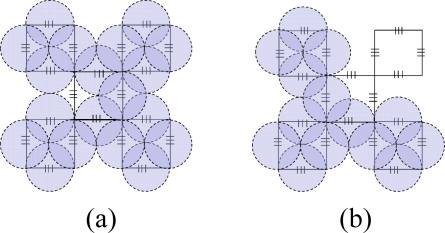
**(a)** Area loss in case 1; **(b)** Area loss in case 2.

After recovery, only one FBG sensor node cannot be recovered in case 1, as shown in the shadow ellipse in Figure 11, while six FBG sensor nodes cannot be recovered in case 2, as shown in Figure 12. Obviously, the lost coverage area in case 2 is much higher than that of case 1. The lost ratio of the coverage area in case 1 is calculated as shown in Figure 13(a). Similarly, the lost ratio of the coverage area in case 2 is calculated as shown in Figure 13(b). The lost area and lost ratio in case 1 is calculated according to formulas (6) and (7). For the coverage area after recovery, the lost area and the lost ratio in case 2 are calculated according to formulas (8), (9) and (10) respectively.

(6) $$S_{\mathrm{Lost}1}={4r}^{2}-{\pi r}^{2}\approx 0.86r^{2}$$

(7) $$R_{\mathrm{Lost}1}(2)=0.86r^{2}/46.84r^{2}\approx 1.8\%$$

(8) $$S_{\mathrm{Actual}2}=3\times {\left(2r\right)}^{2}+3\times 2\pi r^{2}+2\times \pi r^{2}-5\times \left(\frac{\pi }{2}-1\right)r^{2}\approx 28.27r^{2}$$

(9) $$S_{\mathrm{Lost}2}=46.84r^{2}-28.27r^{2}\approx 18.57r^{2}$$

(10) $$S_{\mathrm{Lost}2}(2)=18.57r^{2}/46.84r^{2}\approx 39.6\%$$

As mentioned previously, there are various combinations of positions of the two link failures. These are as follows: {SSC1 & SSC2, SSC1 & SSC3, SSC1 & SSC4, SSC1& SSC5, SSC2 & SSC3, SSC2 & SSC4, SSC2 & SSC5, SSC3 & SSC4, SSC3 & SSC5, and SSC4 & SSC5}. All 10 combinations can be classified into the above two cases. The important issue is how many should be classified into case 1 and how many should be classified into case 2. They are classified according to the following rules, by considering SSC5 separately. Each rule is exclusive with respect to the others.

**Figure 14.**
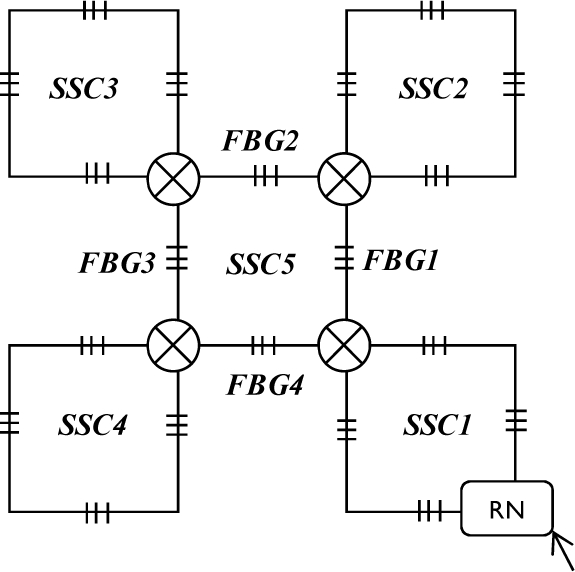
FBG distribution in SSC5.

Rule 1: If one of the two link failures lies in SSC1 which is directly connected to the RN node, the combination is classified into case 1. There are three combinations as follows:
  *Case 1*: {SSC1 & SSC2, SSC1 & SSC3, SSC1 & SSC4}
Rule 2: If the two link failures lie in the two SSCs which are parallel in position besides SSC5, the combination is classified into case 1. There are two combinations as follows:
  *Case 1*: {SSC2 & SSC3, SSC3 & SSC4}
Rule 3: If the two link failures lie in the two SSCs which are diagonal in position, the combination is classified into case 2. There is one combination as follows:
  *Case 2*: {SSC2 & SSC4}
Rule 4: In view of the fact that one link failure lies in SSC5, the combinations cannot be directly classified into case 1 or case 2. If the position of another link failure is separated by two 2 × 2 switches from the one in SSC5, the combination should be classified into case 2, except for the cases when the other link failure lies in SSC1. While if the two positions are only separated by one 2 × 2 switch, the combination should be classified into case 2. There are 10 sub-combinations that can be classified into case 1, and six sub-combinations that can be classified into case 2, as follows (referring to Figure 14):
  *Case 1:* {FBG1 & SSC1, FBG1 & SSC2, FBG2 & SSC3, FBG2 & SSC4, FBG3 & SSC3, FBG 3 & SSC4, FBG4 & SSC1, FBG 4 & SSC4, FBG2 & SSC1, FBG3 & SSC1}
  *Case 2:* {FBG1 & SSC3, FBG1&SSC4, FBG2 & SSC4, FBG3 & SSC2, FBG4 & SSC2, FBG4 & SSC3}

However, the sub-combinations came from the four combinations between SSC5 and the other SSCs (SSC5 & SSC1, SSC5 & SSC2, SSC5 & SSC3, and SSC5/4). Therefore, a total of 2.5 combinations are classified into case 1, and 1.5 combinations are classified into case 2, according to rule 4. So when two link failures happen, there are three combinations according to rule 1, two combinations according to rule 2, and 2.5 combinations according to rule 4, which can be classified into case 1. So, a total of 7.5 combinations are classified into case 1, and 2.5 combinations are classified into case 2. Considering that the loss ratios in case 1 and case 2 are 1.8% and 39.6% respectively, the availability when two link failures happen, says *A_2_*, can be calculated by formula(11) as:

(11) $$A_{2}=1-\frac{P_{2}\times R_{\mathrm{Lost}1}(2)\times (\#\mathrm{ofcase}1)+P_{2}\times R_{\mathrm{Lost}2}(2)\times (\#\mathrm{ofcase}2)}{\left(\#\mathrm{ofcase}1)+(\#\mathrm{ofcase}2\right)}=1-0.1125P_{2}$$

#### (1) Three link failures

**Figure 15.**
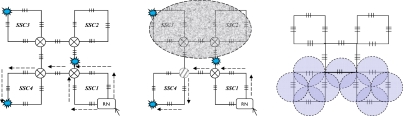
Sensing coverage under three failures.

After discussing the network reliability in case of one link failure and two link failures, the reliability when three link failures happen is discussed. However, only the worst case is discussed. When three link failures happen, the worst case of losing sensing coverage happens in the situation shown in Figure 15. After recovery, it is still the case that more than half of the FBG sensors cannot be recovered. If three link failures happen, the actual coverage is shown in Figure 15. The actual coverage, the lost coverage, the lost ratio and the availability when three link failures happen, denoted by *A*_3_ are calculated according to formulas (12), (13), (14), and (15), respectively.

(12) $$S_{\mathrm{Actual}}=2\times {\left(2r\right)}^{2}+2\times 2\pi r^{2}+\pi r^{2}/2+\left(1-\pi /4\right)r^{2}\approx 18.345r^{2}$$

(13) $$S_{\mathrm{Lost}}=46.84r^{2}-18.35r^{2}\approx 28.49r^{2}$$

(14) RLost(3)=28.49r246.84r2≈60.8%

(15) $$A_{3}=1-P_{3}\times R_{\mathrm{Lost}}(3)=1-P_{3}\times 60.8\%=1-0.608P_{3}$$

#### (2) Reliability calculation

As the probability of more than three link failures happening simultaneously is quite low, this is not considered. This paper considers the network availabilities supposing the probabilities of one link failure to be 0.1%, 0.05%, and 0.01% respectively. The probabilities of *i* (1, 2, 3) link failures within one or multiple SSCs are shown in Table 1. They are calculated according to formulas (2) and (3). The availabilities of sensing coverage in case of *i* link failures are calculated according to formula (4) and are shown in Table 2.

### 6.2. Scalability Analysis

After analyzing the reliability in case of link failures for the SSC-based SSN, this section analyzes the cost when scaling the sensor networks to large scales. The required numbers of FBGs and switches for both SSC- and PSC- based SSNs are calculated in Tables 3 and 4. As the current technology for embedding FBG sensors can multiplex as more as 1,000 FBGs into one fiber [7], we consider to scale them to multiplex as more as 1,000 or at least close to 1,000 FBGs into one fiber. Thus, the scaling degree of the two kinds of AOFSN is considered to three (the number of FBGs will exceed the 1,000 if further scaled). The calculation of the needed number of sensors and switches after scaling can be calculated as shown in 1) and 2), where *i* means the scaling degree.

1. Number of total sensors and switches for SSC-based AOFSN: $\sum_{s=1}^{s=i+1}4^{s}$ and $\sum_{s=1}^{s=i}4^{s}$.
2. Number of total sensors and switches for PSC-based AOFSN: 5*^i^*^+1^ and $\sum_{s=1}^{s=i}5^{s}$.

The formulas in 1) can be obtained from Figure 8(a) to Figure 8(c). The number of required FBGs and switches in Figure 8(a) are 4 and 1, respectively; after the first scaling as shown in Figure 8(b), the number of required FBGs and switches becomes 4 × 4 + 4 (=20) and 4 respectively; after the second scaling as shown in Figure 8(c), the number of required FBGs and switches becomes (4 × 4 + 4) × 4 (=84) and 4 × 4 + 4 (=20) respectively. Inductively, the number of required FBGs and switches for the SSC-based AOFSN are $\sum_{s=1}^{s=i+1}4^{s}$ and $\sum_{s=1}^{s=i}4^{s}$ respectively. The formulas in 2) for PSC-based AOFSN can be obtained from Figure 9(a) to Figure 9(c) by the same induce as that for SSC-based AOFSN.

Tables 3 and 4 show the scaling speed and the scaling cost in terms of the required numbers of FBGs and switches for SSC and PSC-based SSNs according to the above calculation. Moreover, the sensing coverage for different scaling degrees is also shown. In the context of SSC-based SSNs, the number of sensor nodes increases from 20 to 84 when scaling from degree 1 to degree 2, while the needed number of switches only increases from 4 to 20. When it is scaled to degree 3, the number of required switches is increased to 84, which is quite small compared to the number of required 340 FBG sensors. This is indicating good scalability in view of the large number of FBGs after scaling, and less cost in view of the number of switches compared to that the corresponding FBGs. In view of the PSC-based SSN, the scaling speed in terms of the number of FBGs is better than that of the SSC-based SSN; however, the cost in terms of the number of required switches is much higher than that of the SSC-based SSN.

## 7. Conclusions

This paper considered analyzing the reliability and scalability of a large-scale hierarchical all optical fiber sensor networks based on FBG sensors. The Hierarchical AOFSN consists of three levels to guarantee reliability. We focused on the third sensor subnet level due to its passivity. To guarantee reliability for the first two higher levels, the switch architecture for RN was proposed. This kind of RN architecture is recoverable for the SSN system when link failure happens between RNs and InS or between FBGs, or both. The basic segment in the third SSN level is called the sensor cell, including square-based SC (SSC) and pentagon-based SC (PSC) in our research. The two kinds of SCs were proposed based on their regularity and unicursal characteristic, which is good for simple routing and good scalability. The reliability of SSC-based AOFSN was evaluated and analyzed in terms of the sensing ability (coverage area) in cases that one or multiple link failures happen. And the sensing reliability of the proposed architecture even exceeds 99.999%, satisfying the five 9s’ requirement of reliability. The scalability was evaluated in terms of the maintenance of a self-similar topology as well as the scaling speed of sensing coverage after the SSC- and PSC-based AOFSNs have been scaled. The cost in terms of the required number of FBGs and 2 × 2 switches was also considered as an evaluation of the scalability. The calculations show that the rate of increase of the number of required FBGs is much higher than that of the corresponding number of required 2 × 2 switches and the sensing coverage is also scaling fast.

## Figures and Tables

**Table 1.** Probability of *i* link failures: *P_i_*

***P***	***0.1%***	***0.05%***	***0.01%***
***P******_i_***			
***P_1_***	0.019624	0.009905	0.001996
***P_2_***	0.000157	3.96E-05	1.6E-06
***P_3_***	6.29E-07	7.93E-08	6.39E-10

**Table 2.** Network Availability of *i* link failures: *A_i_*

***P***	***0.1%***	***0.05%***	***0.01%***
***A_i_***			
***A_1_***	1	1	1
***A_2_***	0.99996	0.999996	1
***A_3_***	0.999999	1	1

**Table 3.** Scalability of SSC.

**Scaling Degree**	**i = 1**	**i = 2**	**i = 3**
**SSC**			
**# of sensors**	20	84	340
**# of switches**	4	20	84
**Sensing Coverage**	10.28*r*^2^	46.84*r*^2^	193.08*r*^2^

**Table 4.** Scalability of PSC.

**Scaling Degree**	**i = 1**	**i = 2**	**i = 3**
**PSC**			
**# of sensors**	25	75	625
**# of switches**	5	30	155
**Sensing Coverage**	14.43*r*^2^	61.33*r*^2^	311.00*r*^2^
